# Nutritional Safety and Health Resilience in Europe: Declining Micronutrient Intakes, Widening Distributional Disparities and Persistent Inadequacies Revealed from National Dietary Surveys

**DOI:** 10.3390/nu18172860

**Published:** 2026-09-02

**Authors:** Samantha Christie, Adrian F. Gombart, Thomas R. Hill, Tao You, Philip C. Calder

**Affiliations:** 1 Greenwich Integrated Health, London SE10 8SR, UK; 2 Department of Biochemistry and Biophysics, Linus Pauling Institute, Oregon State University, Corvallis, OR 97331, USA; adrian.gombart@oregonstate.edu; 3 Human Nutrition and Exercise Research Centre, Population Health Sciences Institute, Faculty of Medical Sciences, Newcastle University, Newcastle upon Tyne NE1 7RU, UK; 4 Department of Sport Science and Nutrition, Maynooth University, Maynooth, W23 C6T0 County Kildare, Ireland; 5 Department of Pharmacology and Therapeutics, University of Liverpool, Liverpool L69 7ZX, UK; tao.you@liverpool.ac.uk; 6 Centre of Excellence for Long-Acting Therapeutics (CELT), University of Liverpool, Liverpool L69 7ZX, UK; 7 Beyond Consulting Ltd., 14 Tytherington Park Road, Macclesfield SK10 2EL, UK; 8 School of Human Development and Health, Faculty of Medicine, University of Southampton, Southampton SO16 6YD, UK; pcc@soton.ac.uk; 9 NIHR Southampton Biomedical Research Centre, University Hospital Southampton NHS Foundation Trust and University of Southampton, Southampton SO16 6YD, UK

**Keywords:** micronutrient, vitamin, trace mineral, intake, inadequacy, recommendations, temporal changes, European, nutritional safety

## Abstract

**Background:** Nutritional safety has traditionally focused on preventing excessive nutrient exposure, yet chronic micronutrient inadequacy may also impair physiological function and health resilience. As the European Commission prepares to harmonise maximum permitted levels for food supplements and fortified foods, understanding long-term population intake trends is increasingly important. We assessed changes in micronutrient intakes and adequacy among adults in four European countries. **Methods:** Repeated, cross-sectional, secondary analyses of published adult (≥18 years) micronutrient intakes over approximately two decades were conducted using publicly available, non-harmonised national dietary surveys at non-contemporaneous time points in the Netherlands, Ireland, France and the United Kingdom (*n* = 17,261). Mean, lower-tail (5th centile; UK 2.5th) and upper-tail (95th centile; UK 97.5th) intakes were evaluated against dietary reference values, and changes in population intake estimates over the past two decades were examined. **Results:** Intakes of several micronutrients declined substantially, with mean and lower-tail intakes of vitamins D and A, riboflavin, folate and of calcium, iron and iodine decreasing by up to 50% in some population groups, indicating widening intake disparities and increasing risk of chronic insufficiency. Although improvements in some micronutrients were consistent with fortification policies and dietary changes, inadequacies remained widespread, including for vitamin D across all countries, iron in women, potassium in UK women, selenium in most populations and copper in Irish women. Folate intake in UK women and vitamin A intake in France were marginal. **Conclusions:** Declining intakes of several micronutrients and persistent inadequacies indicate emerging nutritional safety concerns in Europe. Population intake trends and chronic insufficiency should be explicitly considered in nutritional safety assessments, maximum permitted level discussions and modelling and future public health policy.

## 1. Introduction

National food surveys are an important resource for informing nutrition policy and guiding public health and safety strategies. Cross-sectional surveys consistently demonstrate insufficient intakes of multiple vitamins and minerals across many European populations [1,2,3,4,5]. However, temporal analyses examining how intakes of different micronutrients have changed over time remain limited. Existing evidence reveals that despite decades of dietary guidance, intakes of several vital micronutrients have declined or remained suboptimal in some European countries, including the UK [6,7,8,9,10].

**Figure 1:**
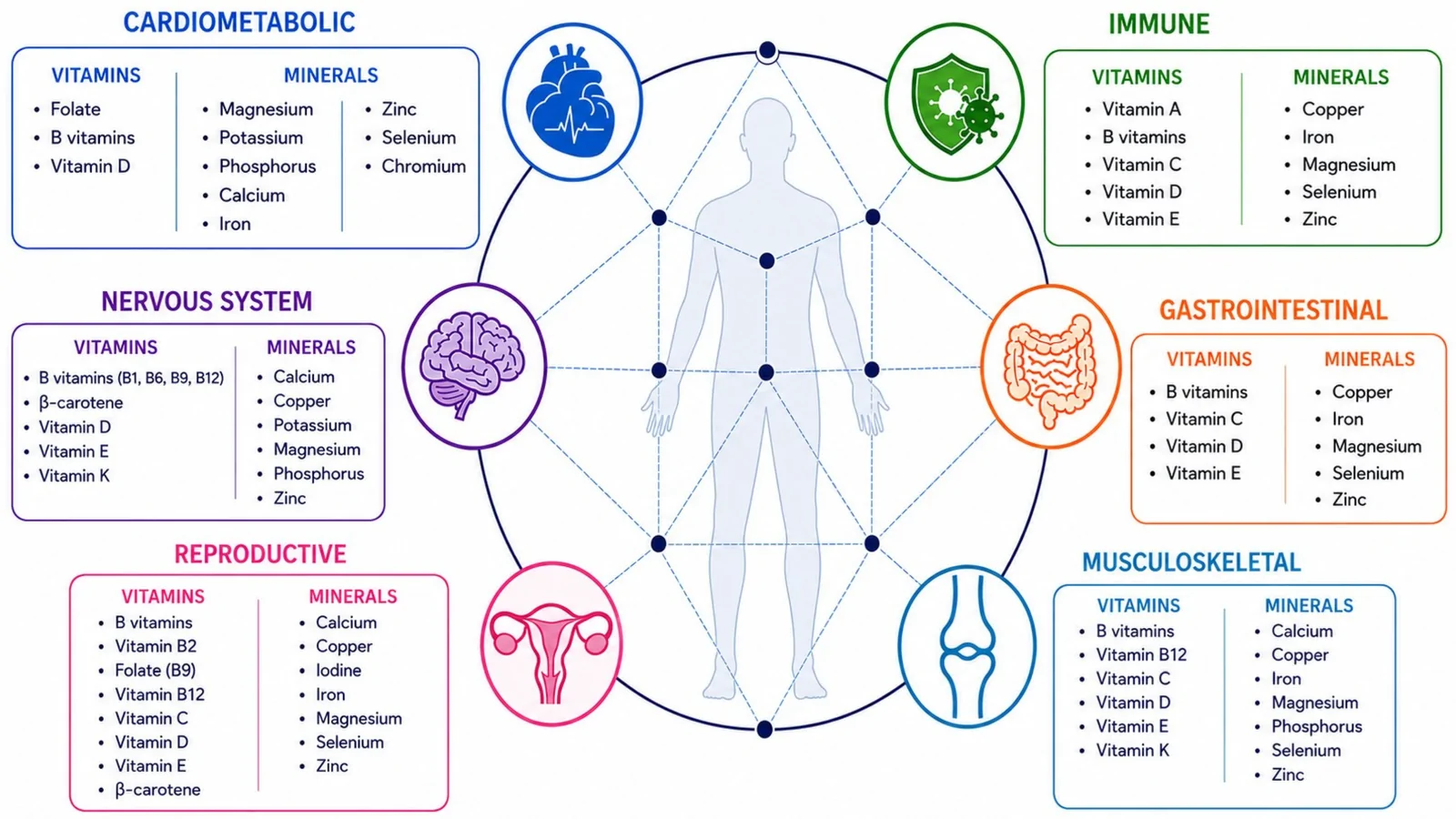
Important micronutrients for organ system function. The figure shows the vitamins and minerals essential for six major organ systems (selective and non-exhaustive): brain and nervous system, reproductive, immune, musculoskeletal, gastrointestinal, and cardiometabolic. Organ system interconnectedness from the bidirectional cross-talk from the gastrointestinal tract (enteroendocrine, lymphoid and microflora) to all other body systems (dotted blue lines). Each category lists the relevant vitamins (e.g., B-complex, C, D, E, K, β-carotene) and minerals (e.g., Ca, Cu, Fe, Mg, Se, Zn) required for normal function of that system. Visual design and graphical presentation were developed with assistance from ChatGPT (GPT-5.5 Instant, OpenAI); scientific content was determined and verified by the authors.

Micronutrient inadequacies are thought to reflect broader shifts in dietary habits, reduced diet quality and poor adherence to healthy eating guidelines [6,7]. Given that multiple micronutrients are required to support the optimal functioning of multiple physiological systems (Figure 1), sustained inadequate intake may progressively impair physiological function and resilience and contribute to increased disease burden and health risks, even in the absence of overt deficiency disease [11,12,13,14,15]. Table 1 summarises the documented associated adverse effects of various micronutrient insufficiencies on specific organ and physiological systems. While severe deficiency disorders such as scurvy are relatively uncommon in Europe, aside from nutritional rickets and osteomalacia [16,17,18], chronic marginal insufficiency may still adversely affect metabolic, musculoskeletal, nervous system, gastrointestinal, reproductive and immune function across the life course [10,19,20,21,22,23,24,25,26] (Table 1). Such effects may be particularly relevant to vulnerable groups with increased nutritional requirements or dietary inadequacy.

Micronutrients are increasingly recognised as integral components of biological systems that maintain a network of dynamic, physiological homeostatic processes [10,19,27]. Emerging evidence suggests that chronic micronutrient insufficiency may not simply result in isolated nutrient-specific effects, but can disrupt interconnected regulatory networks involved in surveillance, inflammatory control, epithelial barrier integrity, cell signalling and energy metabolism (Table 1). These disturbances may remain clinically silent for prolonged periods, yet reduce physiological resilience, impairing the body’s capacity to respond effectively to infection, injury, environmental stressors and metabolic challenges [7,20]. This systems-based perspective has shifted attention beyond the prevention of classical deficiency disease towards understanding how persistent low intakes may influence population health through cumulative, interdependent network physiology, with effects on multiple systems and shared inflammatory pathways [10,12,15,28]. Consequently, assessment of nutritional safety may require consideration of both overt deficiency and the broader, functional consequences of chronic insufficiency across the distribution of habitual dietary intakes.

Importantly, the consequences of inadequate micronutrient intake may not be distributed uniformly across populations. Individuals with intakes either near or below reference intakes or in excess of thresholds may experience disproportionate reductions in physiological reserve and adaptive capacity, even when population mean intakes remain largely adequate [29,30,31]. As a result, changes occurring at the lower end of intake distribution may have a greater public health significance than changes in central tendency alone. This perspective is particularly relevant where intake distributions widen over time, increasing the proportion of individuals exposed to chronic insufficiency. Nutritional safety therefore encompasses not only protection against excessive exposure but also consideration of population cohorts whose habitual intakes may be insufficient to support normal physiological function.

Current food safety frameworks have primarily focused on preventing excessive micronutrient intakes. This leads to the tolerable safe upper intake levels (ULs) and associated scientific risk management approaches for food supplements, which include dietary intakes of fortified and non-fortified foods [32,33,34,35,36,37]. These also account for changes in micronutrient intake distributions over time within the legal status of food supplements [38]. The European Food Safety Authority (EFSA) uses national dietary intake data as part of the scientific framework underpinning these assessments [39,40,41,42]. Within this context, nutritional safety can be viewed as the maintenance of habitual micronutrient intakes within a range that supports and maintains physiological functionality, resilience and homeostasis across the population while minimising risks associated with both deficiency and excess.

Given the changing dietary patterns, we explored the hypothesis that micronutrient intakes in European adults have not improved over time and remain inadequate relative to established dietary reference values. If this is the case, then persistent inadequacy warrants careful consideration within ongoing discussions regarding regulatory restrictions on food supplements and fortified foods. If confirmed, persistent inadequacies may have implications for ongoing discussions regarding Maximum Permitted Levels (MPLs) for food supplements and fortified foods, particularly in populations already characterised by low habitual micronutrient intakes. 

This study examined prospective changes in micronutrient intakes among adults over the past two decades. We conducted secondary analyses of consecutive, publicly available national dietary surveys from countries where consistent methodology across survey waves also met our wider inclusion criteria. Surveys from Ireland, the Netherlands, France and the UK were subsequently analysed. We evaluated temporal changes in micronutrient intake distributions and assessed the extent to which recent intakes met established dietary reference values, including Estimated Average Requirements (EARs), Lower Reference Nutrient Intakes (LRNIs) and Reference Nutrient Intakes (RNIs). Additionally, we considered these findings in relation to forthcoming regulatory changes from the European Commission for MPLs for food supplements and fortified foods.

## 2. Materials and Methods

### 2.1. Data Sources and Selection Criteria

We examined repeated, cross-sectional, publicly available national dietary datasets to assess micronutrient intake estimates across European populations. Consistent with EFSA’s European National Food Survey Summary recommendations for dataset selection, voluntary and mandatory fortified foods were considered integral to the habitual diet [43]. We also adhered to EFSA’s guidance regarding utilisation of the highest quality, nationally representative data for evaluating diet–health relationships [39,40]. We studied countries where comparative surveys were publicly available, which limited our secondary analysis to the Netherlands, Ireland, France and the United Kingdom.

### 2.2. Inclusion Criteria for National Food Surveys

- Nationally representative, individual level food consumption data excluding household budget surveys.
- Repeated surveys within the same country.
- Ethical approval in accordance with the Declaration of Helsinki and relevant institutional review boards.
- Participants ≥18 years of age.
- Consistent methodology across survey years.
- Dietary intake data based on food sources only, including fortified foods but excluding food supplements.
- Consistent mean or median intakes available across all countries. Percentile intakes available where possible. The lowest percentile or lower tail was defined as the 5th percentile (p5) for NL and IR and 2.5th (p2.5) for the UK. The highest percentile or upper tail was defined as the 95th percentile (p95) for NL and IR, and 97.5th (p97.5) for UK.

### 2.3. Included Datasets and Population Demographics

Four European countries met the inclusion criteria: the Netherlands, Ireland, France, and the UK. Another 17 countries published dietary surveys but did not have comparable repeated measurements. These were excluded from the study. The national dietary surveys analysed were:

(i) The Netherlands (NL): Dutch National Food Consumption Survey (DNFCS) 2012 and 2021 [44,45];
(ii) Ireland (IR): North/South Ireland Food Consumption Survey (NSIFSC 1997) and National Adults Nutrition Survey (NANS 2010) [46,47];
(iii) The United Kingdom (UK): National Diet and Nutrition Survey (NDNS) 2003, 2014 and 2020 [48,49,50];
(iv) France (FR): INCA2 (2006–2007) and INCA3 (2013–2014) [51,52].

The UK NDNS 2019–2023 rolling programme was excluded due to methodological changes in dietary assessment, specifically the transition to the web-based, Intake-24^®^ system, which precluded direct comparability with earlier surveys. Across the included surveys, the total sample size was 17,261 nationally representative adults. Analyses were conducted separately by sex where data permitted. The interval between field survey years ranged from 8 years (France) to 18 years (UK; Table 2). Survey fieldwork spanned from 1997 (Ireland) to 2021 (Netherlands). The number of micronutrients assessed varied by country, ranging from 12 (UK) to 18 (Netherlands).

### 2.4. Micronutrient Intake Data Handling

Micronutrient intake data were obtained from published summary reports for observed, unadjusted, repeated national dietary surveys and analysed descriptively within countries over time. Mean intakes were examined for all countries, since median intakes were not consistently reported across nations, with lower and upper tail intake distributions assessed using reported percentiles where available: p5 and p95 for the Netherlands and Ireland, and p2.5 and p97.5 for the UK. Lower and upper tail intake distributions were not available from French surveys. Given differences in survey methods and reporting formats, the analysis was intended primarily to characterise within-country temporal changes rather than fully harmonised cross-country comparisons. Micronutrients with inconsistent reporting or recognised methodological limitations were excluded from relevant analyses.

### 2.5. Data Analysis

Based on published summary statistics, we examined within-country changes in mean micronutrient intakes and, where available, lower and upper percentile intakes across repeated survey waves. Percentage change was used to characterise the direction and magnitude of change across the intake distribution. No formal statistical testing was performed. Percentage changes were assessed between the first and last survey years across countries, aside from vitamin A and selenium in the UK. Here, the intermediate year (2014) was compared to the latest survey (2020), since retinol equivalents and selenium were consistently assessed across these two UK survey waves, in line with the other countries.

Changes of 10% or more were considered notable for pragmatic purposes, and distinct from statistically significant or other inferential descriptors. Cross-country comparisons were interpreted cautiously because survey methods and reporting differed.

### 2.6. Micronutrient Adequacy Assessment

Micronutrient intakes were evaluated relative to the established daily dietary reference values (UK) [53]. For vitamins A, B2 (riboflavin), B9 (folate), B12 and C, and minerals calcium, magnesium, iron and zinc, Estimated Average Requirements (EARs) were pre-defined. For these micronutrients, mean intakes from the most recent national food surveys were expressed as a percentage of the EAR. For potassium, iodine and selenium, approximated Median Intake Values (aMIVs) were applied. The approximated Mean Intake Values (aMIVs) for selenium and iodine were calculated using a log transformation, since dietary intakes of these trace minerals in the UK do not conform to a normal Gaussian distribution [54,55].

(1) $$\ln\left(P50\right)=\frac{\ln\left(P2.5\right)+\ln\left(P97.5\right)}{2}$$

Therefore,

(2) $$P50=\exp\left(\frac{\ln\left(P2.5\right)+\ln\left(P97.5\right)}{2}\right)$$

where P2.5, P50, and P97.5 were the 2.5th, 50th and 97.5th percentiles, respectively.

Potassium intake is approximately normally distributed. Hence, the potassium aMIV was calculated by the following: $P50=0.5\times \left(P2.5+P97.5\right)$ [56].

For vitamin D and copper, intakes were compared to the Reference Nutrient Intakes (RNIs), in line with recommendations from the Scientific Advisory Committee on Nutrition (SACN, UK) [53,57]. At the distribution extremes, lower percentile intakes were expressed as a percentage of the Lower Reference Nutrient Intake (LRNI), whereas upper percentile intakes were expressed relative to the RNI. All analyses were conducted separately for men and women where data were available.

### 2.7. Figure Development

Figure 1 was developed to produce a schematic representation of selected micronutrients of relevance to the normal function of six major organ systems in the body. The scientific content, including the selection and categorisation of micronutrients and organ systems, was determined and verified by the authors. Chat GPT (GPT-5.5 Instant, OpenAI, San Francisco, CA, USA) was used as a generative AI-assisted design tool to aid the visual conceptualisation, organisation and graphical presentation of the figure, including the arrangement of organ-system categories, icons, text and connecting elements. The figure underwent iterative author-led refinement and review. The authors verified the scientific content and approved the final figure. Generative AI was not used to generate or validate the underlying scientific evidence.

## 3. Results

### 3.1. Micronutrient Intake Shifts and Adequacy Across the Distribution

**Figure 2:**
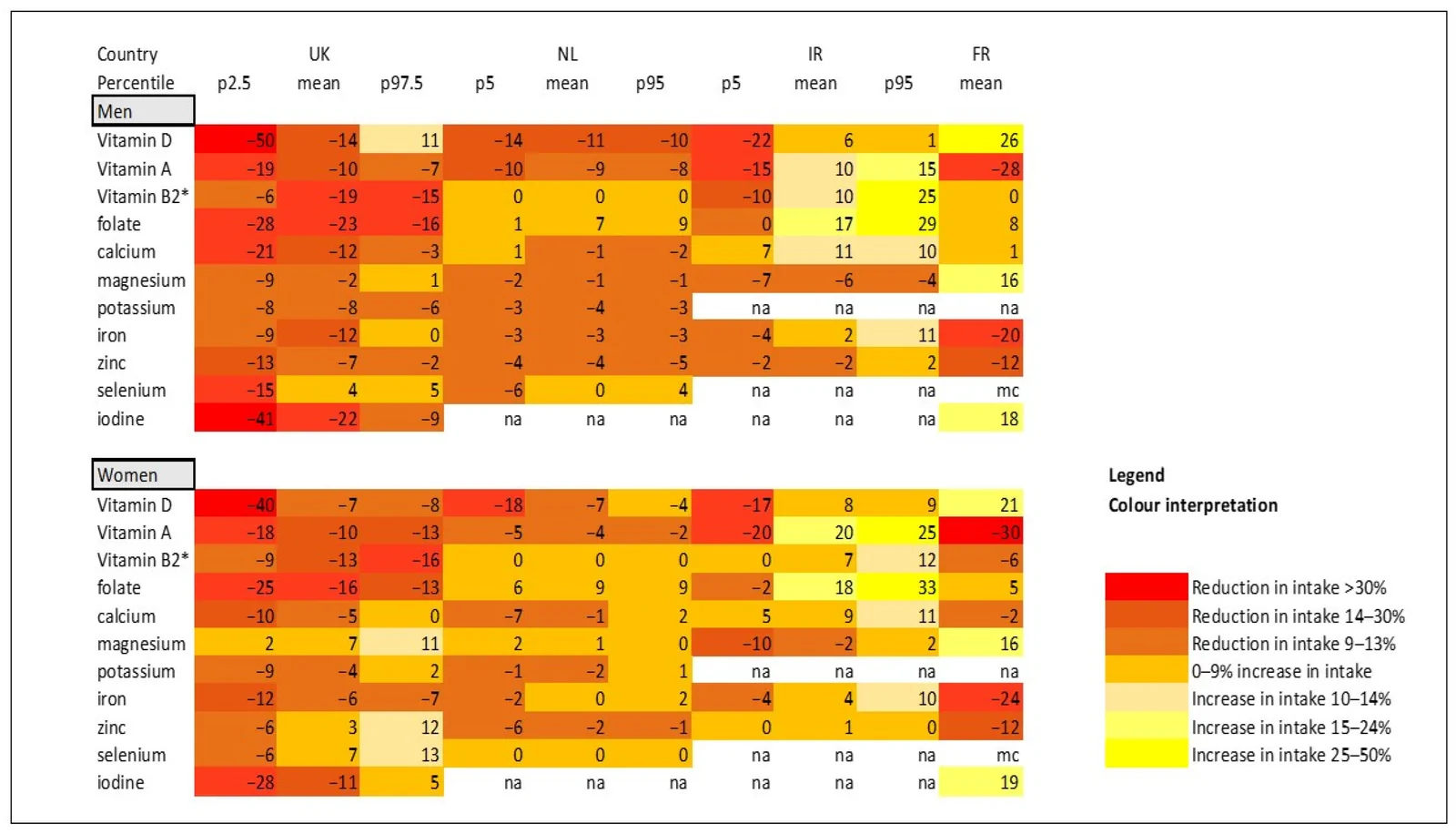
Heatmap summarising percentage changes in micronutrient intakes across percentiles in selected European countries (UK, NL, IR and FR^θ^) ^†^°. Comparisons are latest survey versus previous survey comparisons, aside from UK vitamin A and selenium intake comparisons (2014 vs. 2020). Key: ^θ^: France: mean datasets only; ^†^: vitamin A as Retinol Equivalents (RE); °: food-only datasets, excluding food supplements; *: riboflavin; FR: France; IR: Ireland; mc: methodological concerns; na: not assessed; NL: the Netherlands; P2.5/5: lowest mean 2.5th/5th percentile; P95/97.5: 95th/97.5th percentile; UK: United Kingdom; Vitamin C not assessed separately for men and women.

Changes in micronutrient intakes ≥10% were examined across the intake distribution, with particular focus on whether shifts observed in mean intakes were accompanied by disproportionate deterioration at the lower end of the distribution (Figure 2; combined datasets; Supplementary Materials Figures S1–S6). Overall, the cross-sectional adequacy data indicate that while some mean intakes remained close to, or above, reference requirements, the lower tail of the distribution showed deterioration and more frequent shortfalls below reference thresholds over time. In other words, those already consuming the least micronutrients exhibited increasing nutritional vulnerability.

#### 3.1.1. Vitamins

The lowest intake percentile showed distinctively broader and consistent downward shifts, with no increases in intake observed (Figure 2). The sharpest declines were seen for vitamin D, particularly in the UK (−50% men; −40% women), with smaller but still notable declines in Ireland and the Netherlands (−14 to −22%). Folate intake also declined substantially in the UK (−28 to −25%), while moderate reductions (>10%) were observed across several B vitamins, vitamin C and vitamin A (approximately −10% to −20% across countries).

**Figure 3:**
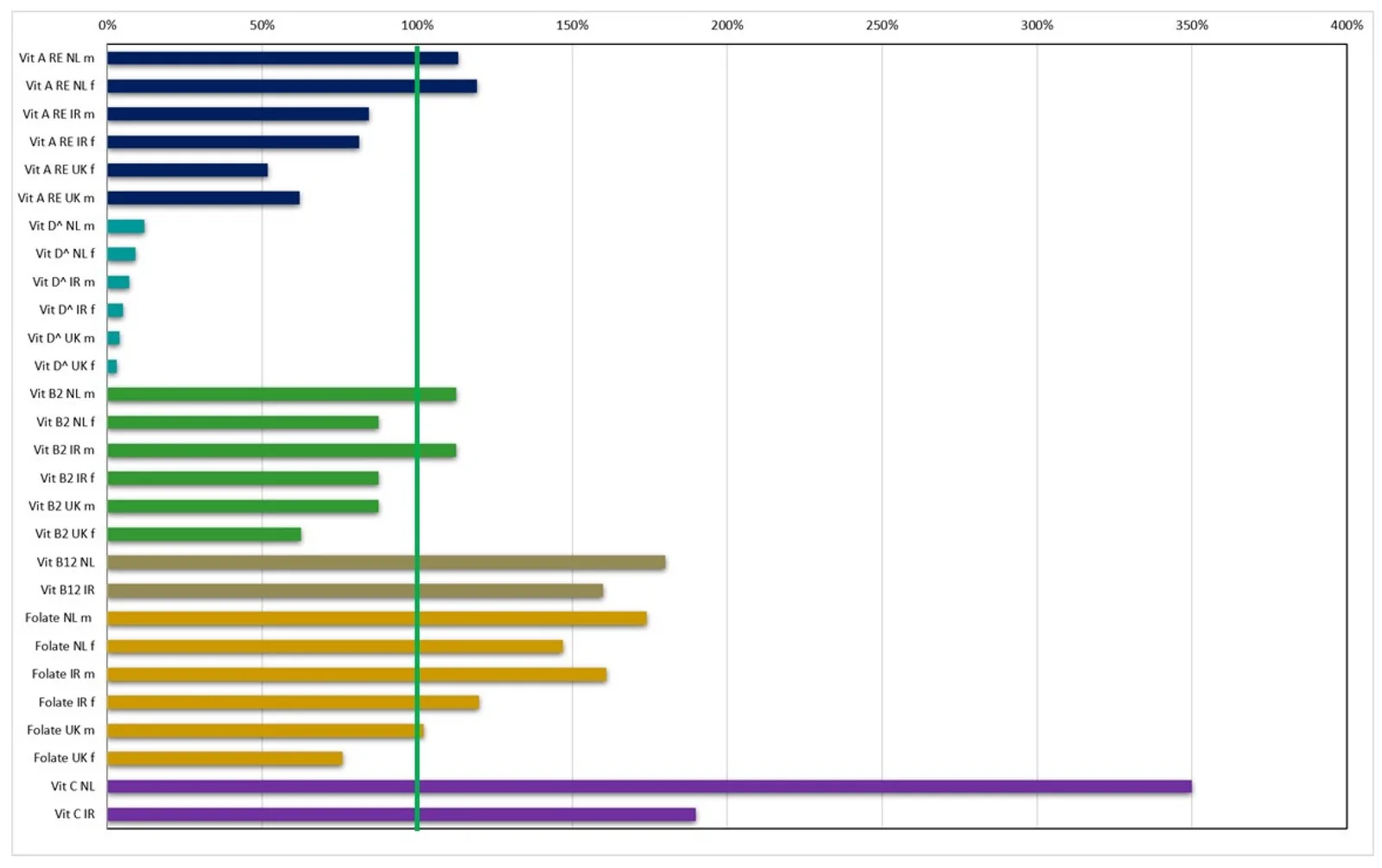
Cross-sectional lower-tail percentile vitamin intakes expressed as a percentage of the Lower Reference Nutrient Intakes (LRNIs) from the Netherlands, Ireland, France and the United Kingdom, straified by sex; latest intake assessed versus Lower Reference Nutrient Intake Key: ^: where no ascribed EAR for vitamin D, comparison made with RNI; f: females; FR: France; IR: Ireland; m: males; NL: the Netherlands; UK: United Kingdom: Vit B2: riboflavin; vitamin B3: niacin. The green vertical line denotes 100% LRNI; horizontal bar colours: dark blue: vitamin A; teal: vitamin D; green: vitamin B2; beige: vitamin B12; yellow: folate and purple: vitamin C.

Cross-sectional intake adequacy comparisons showed that these lower tail declines occurred in groups already close to, or below, adequacy thresholds (Figure 3). Vitamin D intakes at the lowest percentile provided only 3–12% of the RNI across countries, and were substantially lower than mean intakes. Vitamin A intakes in the lower tail fell below the LRNI in Ireland and the UK (UK: 52–62% LRNI; Ireland: 81–84%). Riboflavin intakes were below the LRNI in several groups (63–88%), and UK women’s folate intakes fell below the LRNI (76%). In contrast, vitamin B12 intakes remained above reference levels, even at the lowest percentile (160–180% LRNI).

The largest declines in mean intake were observed for vitamin A (France: −28%; UK: −10% to −30%) (Figure 2). In the UK, mean intakes of folate (−23% men; −16% women) and riboflavin (−19% men; −13% women) exhibited substantial reductions. Moderate declines in mean intake (−10 to 0–14%) were observed across several B vitamins and vitamin D. In contrast, some improvements were evident in Ireland and France, including increases in mean intakes of vitamin D in France (+21 to +26%), folate in Ireland (+17 to +18%) and vitamin B12 in Ireland (−23%).

**Figure 4:**
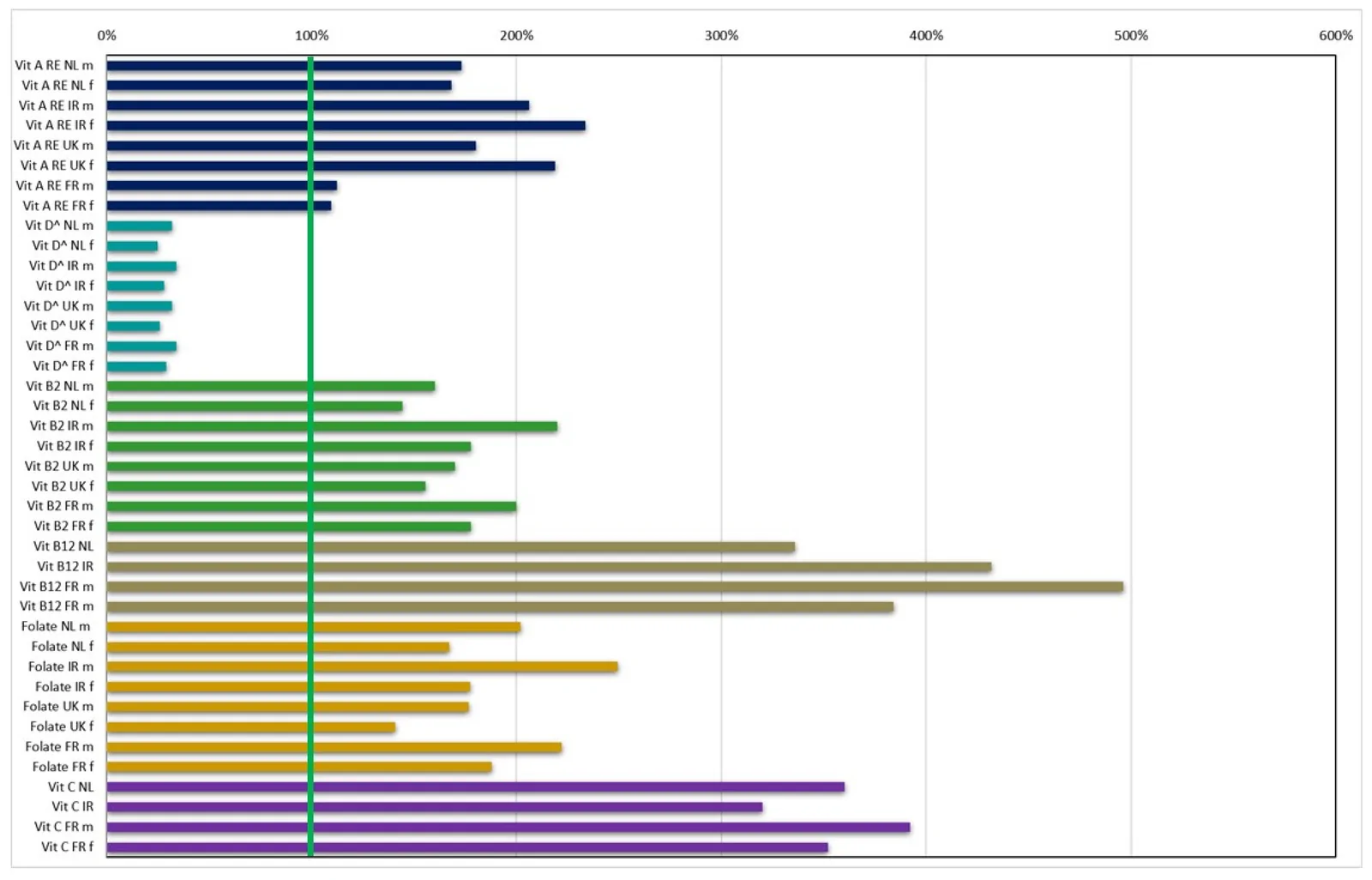
Cross-sectional mean vitamin intakes expressed as a percentage of the Estimated Average Requirements (EARs) from the Netherlands, Ireland, France and the United Kingdom, stratified by sex; latest intake assessed versus Estimated Average Requirement (EAR). Key: aEAR: approximated Estimated Average Requirement; ^: where no EAR ascribed for vitamin D, comparison made with RNI; f: females; FR: France; IR: Ireland; m: males; NL: the Netherlands; RNI: Reference Nutrient Intake. The green vertical line denotes 100% EAR; horizontal bar colours: dark blue: vitamin A; teal: vitamin D; green: vitamin B2; beige: vitamin B12; yellow: folate and purple: vitamin C.

**Figure 5:**
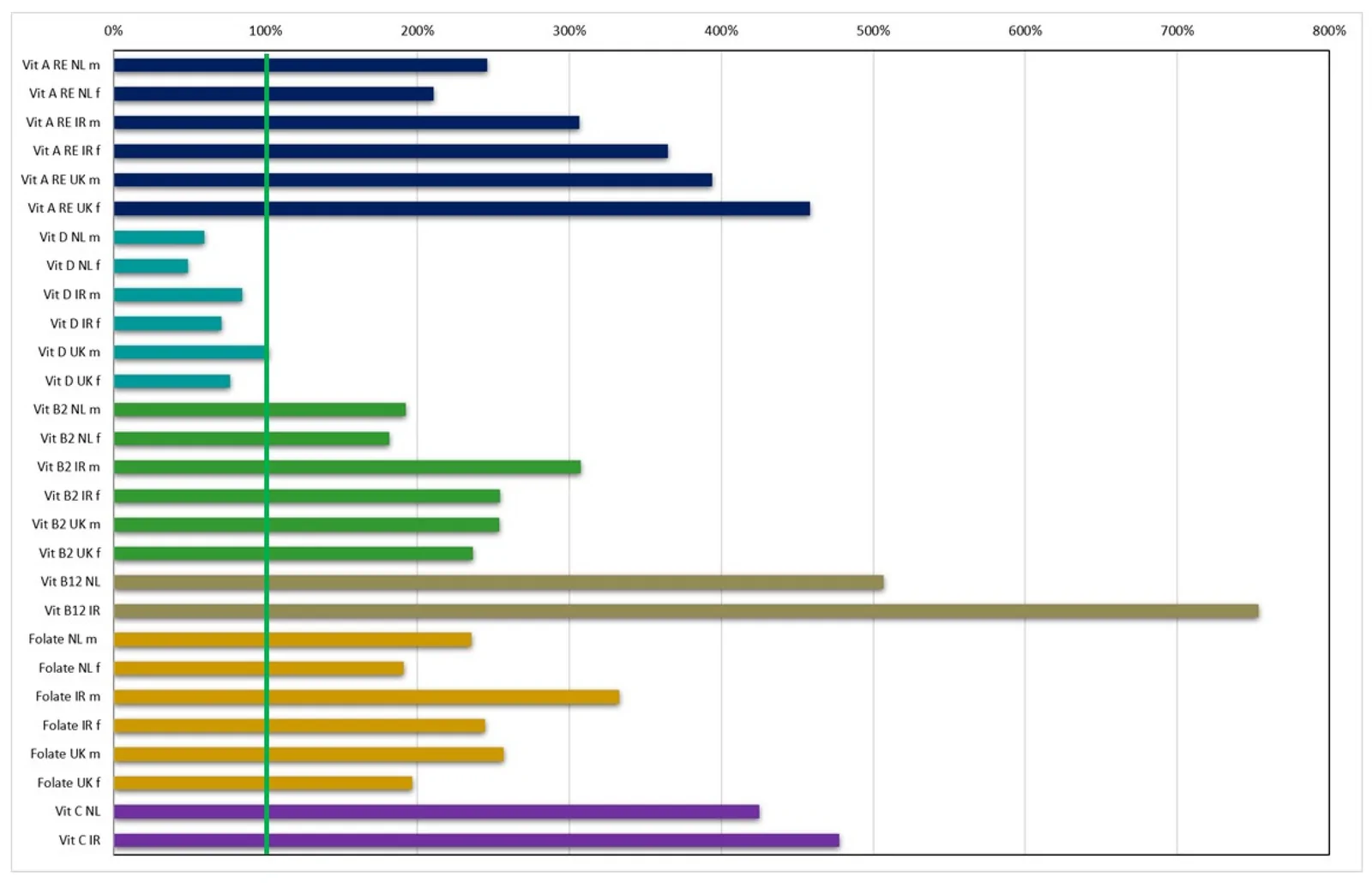
Cross-sectional highest-tail percentile vitamin intakes expressed as a percentage of the Reference Nutrient Intakes (RNIs), stratified by sex; latest intake assessed versus Reference Nutrient Intake. Key: f: females; FR: France; IR: Ireland; m: males; NL: the Netherlands; UK: United Kingdom: Vit B2: riboflavin; vitamin B3: niacin. The green vertical line denotes 100% RNI; horizontal bar colours: dark blue: vitamin A; teal: vitamin D; green: vitamin B2; beige: vitamin B12; yellow: folate and purple: vitamin C.

Despite these changes, mean intakes for most vitamins generally met EARs, including for vitamin A, folate and other B vitamins across countries (Figure 4). This was also broadly the case for intakes at the highest intake percentile (Figure 5). A notable exception was vitamin D, for which no EAR is set; when observed against the RNI (10 µg), mean intakes provided only 25–34% of the recommended intake across countries, indicating persistently low dietary provision even at the population mean. This exception for vitamin D persisted at the highest intake percentile. Vitamin D was the only vitamin at this percentile that failed to reach the RNI across all countries, aside from UK men (102% RNI). Intakes for vitamin D in the Netherlands were the furthest below the RNI for both men and women (60% and 49% of the RNI, respectively). Ireland had similar suboptimal intakes: 85% and 71% RNI for men and women, respectively, at the higher intake percentile.

Taken together, these findings indicate that while mean vitamin intakes often appeared broadly adequate, the lower end of the intake distribution experienced larger declines and more frequent shortfalls below reference thresholds, particularly for vitamin D, vitamin A and folate.

#### 3.1.2. Minerals

**Figure 6:**
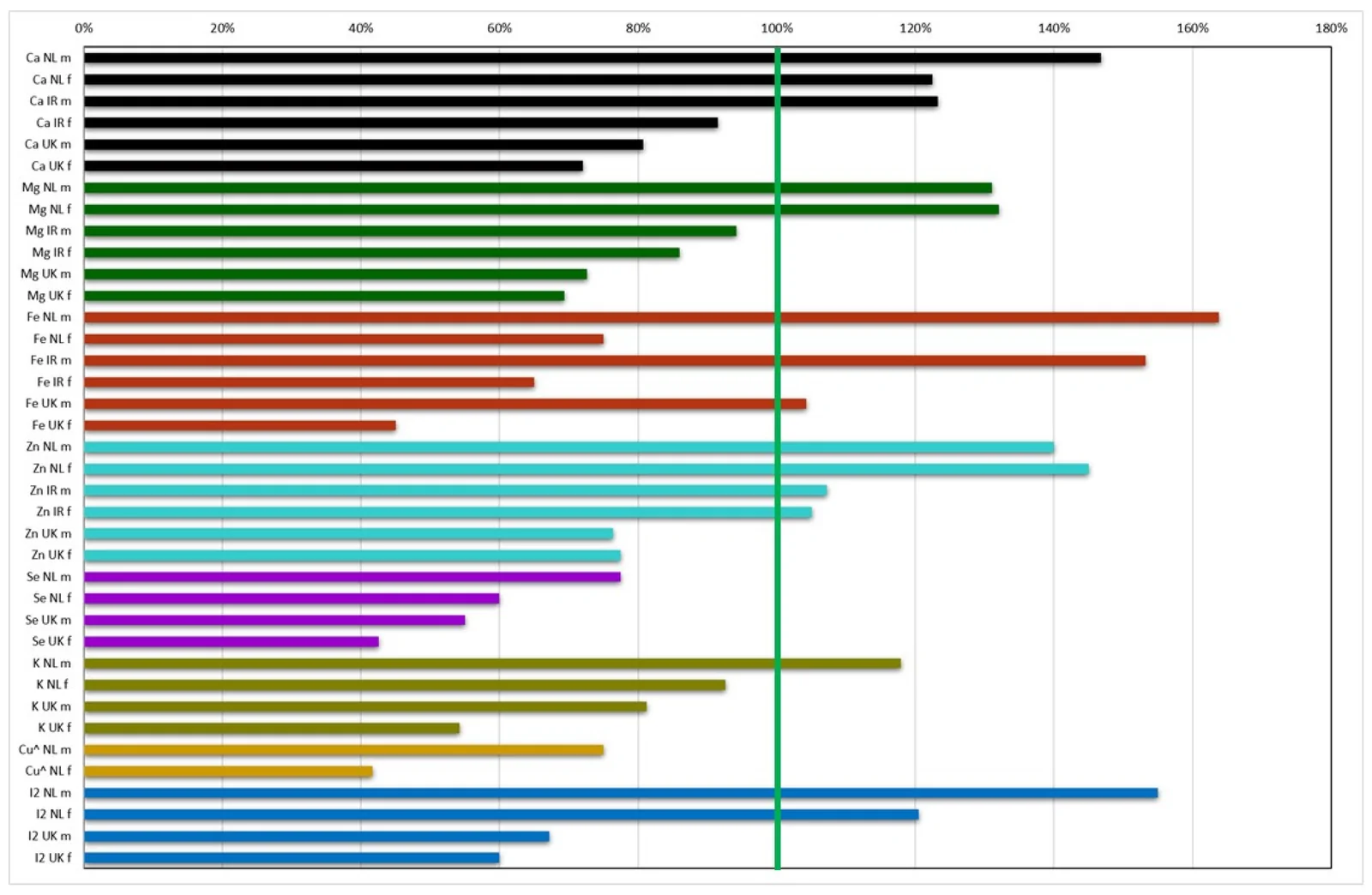
Cross-sectional lower-tail percentile mineral intakes expressed as a percentage of the Lower Reference Nutrient Intakes (LRNIs), stratified by sex; latest intake assessed versus LRNI. Key: ^: where no LRNI ascribed for copper, comparisons made with RNI; Ca: calcium; Cu: copper; Fe: iron; f: females; FR: France; I2: iodine; K: potassium; Mg: magnesium; IR: Ireland; m: males; NL: the Netherlands; Se: selenium; UK: United Kingdom; Zn: zinc. The green vertical line denotes 100% LRNI; horizontal bar colours: black/blue: calcium; dark green: magnesium; red: iron; turquiose: zinc; violet: selenium; olive green: potassium; gold: copper and blue: iodine.

At the lowest intake percentile, changes in mineral intakes showed a similar but more pronounced downward shift, with all notable changes (≥10%) representing declines (Figure 2). The largest reductions were observed for iodine in the UK (−41% in men; −28% in women) and copper in Ireland (−23%), while lower tail intakes of calcium, magnesium, iron, zinc and selenium also declined by approximately 10–21%. These reductions translated into more widespread shortfalls relative to LRNI thresholds (Figure 6). Intakes of calcium, magnesium and potassium fell below the LRNI in several UK and Irish groups, while women’s iron intakes were below the LRNI across countries (45–75%). UK zinc intakes did not meet the LRNI (76–78%), selenium remained below the LRNI in Dutch and UK adults (43–78%), and iodine intakes were particularly low in the UK (60–70% of LRNI). Dutch copper intake also fell below reference levels, particularly among men (42% of LRNI).

**Figure 7:**
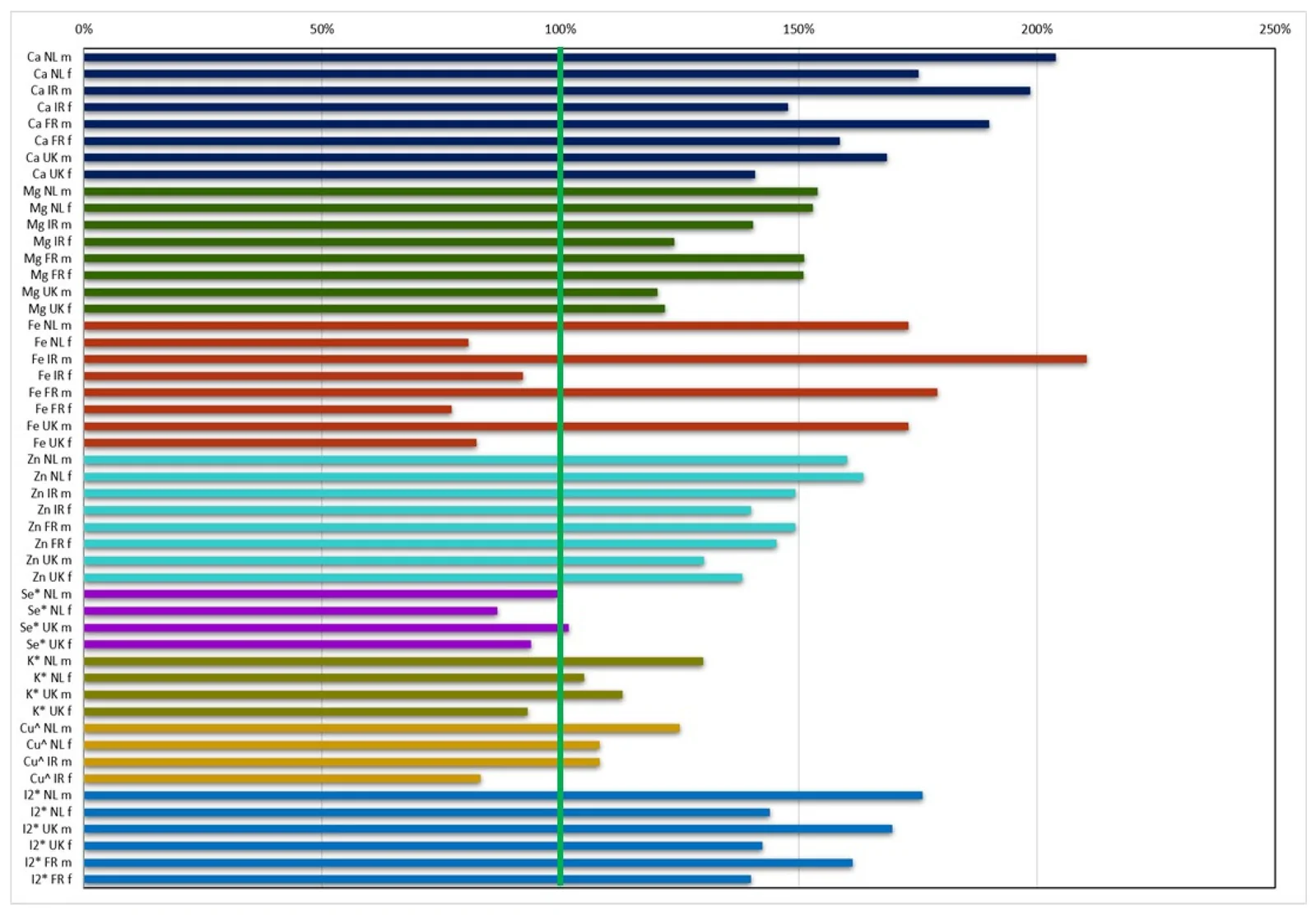
Cross-sectional mean mineral intakes expressed as a percentage of the Estimated Average Requirement (EARs), stratified by sex; latest intake assessed versus Reference Nutrient Intake. Key: *: aMIV: approximated Median Intake Value; ^: where no EAR ascribed for copper, comparisons made with RNI; Ca: calcium; Cu: copper; Fe: iron; f: females; FR: France; I2: iodine; K: potassium; Mg: magnesium; IR: Ireland; m: males; NL: the Netherlands; Se: selenium; UK: United Kingdom; Zn: zinc. The green vertical line denotes 100% EAR or aMIV; horizontal bar colours: black/blue: calcium; dark green: magnesium; red: iron; turquiose: zinc; violet: selenium; olive green: potassium; gold: copper and blue: iodine.

For minerals, changes in mean intakes were again characterised mainly by declines, with fewer increases than observed for vitamins (Figure 7). The largest reductions in mean intake were seen for iron in France (−20% to −24%) and iodine in UK men (−22%), with smaller declines for calcium, zinc and iodine in other groups. Some increases were observed, particularly in France and Ireland, including for magnesium (16%) and iodine (18% to 19%). Comparisons with EARs showed that mean mineral intakes often remained above reference levels for men, but important sex differences emerged. In particular, women’s mean iron intakes were below the EAR across all countries (77–92% of EAR), whereas men’s intakes substantially exceeded the EAR (173–210%). Selenium intakes also remained below aMIV thresholds in both the Netherlands and UK (86–97%), while UK women’s potassium intakes fell below the aMIV (93%). Mean magnesium intakes met the EAR across countries, although UK intakes were lowest relative to other countries.

At the highest intake percentile, changes in mineral intakes were less prevalent and overt than at either the mean or the lowest intake percentile (Figure 2). There were no decreases observed for minerals and there were some increased intakes. These included moderate increases across macro- and trace minerals: specifically, calcium: +10 to +11% in Irish men and women, respectively; magnesium (+11% UK women); iron (+11% and +10% Irish men and women, respectively); zinc (+12%, UK women) and selenium (+13%, UK women).

**Figure 8:**
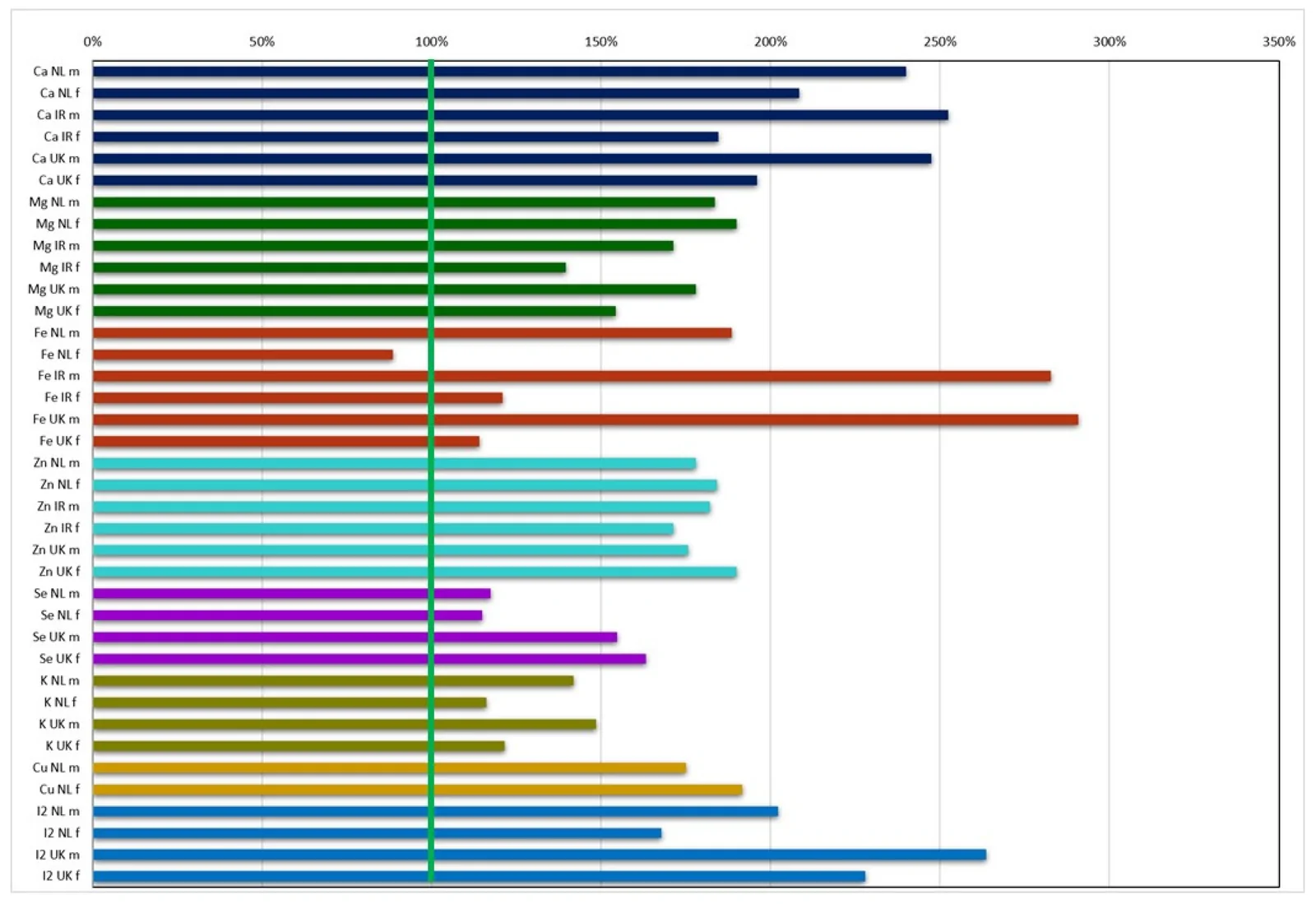
Cross-sectional highest-tail percentile mineral intakes expressed as a percentage of the Reference Nutrient Intakes (RNIs), stratified by sex; latest intake assessed versus Reference Nutrient Intake. Key: Ca: calcium; Cu: copper; Fe: iron; f: females; FR: France; I2: iodine; K: potassium; Mg: magnesium; IR: Ireland; m: males; NL: the Netherlands; Se: selenium; UK: United Kingdom; Zn: zinc. The green vertical line denotes 100% RNI; horizontal bar colours: black/blue: calcium; dark green: magnesium; red: iron; turquiose: zinc; violet: selenium; olive green: potassium; gold: copper and blue: iodine.

Dutch women did not achieve RNI for iron at the highest-tail intakes: iron (89% RNI), whilst UK female intakes just reached the RNI: 114% RNI (Figure 8). Of those mineral intakes close to the RNI, selenium intakes were the more modest, specifically Dutch intakes: 114% RNI for men and 115% RNI for women. Similar marginal intakes were seen for potassium: NL and UK females 116% RNI and 122% RNI, respectively. The highest percentage RNI for minerals was in UK men for iron: 291% RNI.

### 3.2. Summary

Across the intake distribution, mean micronutrient intakes often remained at or above reference levels, which might suggest broadly adequate dietary provision at the population level (Table 3). However, this masked a markedly different pattern at the lower tail of the distribution, where intakes showed larger downward shifts and more frequent failure to meet the LRNI levels. This contrast was particularly evident for vitamin D, where mean intakes already remained low (25–34% of RNI), and lowest-percentile intakes fell to just 3–12% of the RNI. Similar divergence was observed for vitamin A, folate, calcium, magnesium, iron, selenium and iodine, where average intakes often appeared acceptable but lower-tail intakes indicated substantial inadequacy. Overall, these findings suggest that population mean intakes alone may obscure important nutritional safety and vulnerability, with the lower tail of the distribution showing disproportionate deterioration across multiple micronutrients.

## 4. Discussion

### 4.1. Distributional Changes in Micronutrient Intakes Across Populations

When micronutrient intakes were examined across the three intake percentiles, a clear distributional gradient emerged across all four European countries included in this study, with one notable exception. In the UK, folate intake declined across the entire distribution, suggesting a broader population-wide dietary shift, rather than a pattern driven primarily by widening nutritional inequality.

Overall, the most pronounced changes were reductions in intake among individuals at the lowest intake levels, with progressively smaller declines toward the upper end of the intake distribution. These findings raise important concerns regarding both nutritional safety and public health resilience. Declines among those already consuming the lowest levels of micronutrients are likely to increase the prevalence and severity of micronutrient inadequacy, and may reduce physiological resilience at the population level (Table 1). Downward shifts concentrated within nutritionally vulnerable groups therefore represent not only a nutritional safety concern, but also an important public health issue.

### 4.2. Public Health Implications: Nutritional Safety and Inequality

The concentration of declining intakes at the lower end of the intake distribution has important public health implications. Of particular concern is that deterioration occurred among groups whose intakes were already lowest and, for several micronutrients, below reference thesholds. In contrast, changes at the population mean were more heterogeneous, and mean adequacy often remained satisfactory. Consideration of mean intakes alone may therefore obscure deterioration among nutritionally vulnerable segments of the population.

However, interpretation of these findings warrants caution. Individuals with intakes at the lower end of a population distribution do not necessarily have lower physiological requirements. Consequently, comparisons with EARs or LRNIs, while useful at a population level, may not fully capture nutritional vulnerability or resilience within individuals or subgroups. Reliance on these reference values alone may therefore provide an incomplete lens through which to assess public health risk, particularly where population intake distributions are shifting downward across multiple micronutrients simultaneously.

From a population health perspective, the coexistence of declining intakes across several micronutrients suggests that average dietary intake may no longer provide an adequate margin of nutritional safety in some European populations. This concern is amplified where reductions occur concurrently across multiple nutrients because of the reliance on multiple micronutrients in the body’s interconnected homeostatic processes [10,19,28].

The relative preservation of micronutrient intakes at the upper end of the distribution suggests greater nutritional resilience among individuals with higher dietary intakes, potentially reflecting broader dietary diversity, higher overall food consumption, or both.

Taken together, these findings indicate two potentially important patterns: a widening distributional gradient in micronutrient intake, and, for some nutrients, a broader downward shift across the intake distribution. The former concentrates deterioration among those already at greatest risk of inadequacy, potentially exacerabting nutritonal inequality, whereas the latter suggests more widespread changes in dietary exposure. Both patterns have important implications for nutritional safety and population health resilience (Table 1).

### 4.3. The UK Folate Exception and Policy Context

Concerns regarding declining folate intake in the UK have previously been recognised by the Scientific Advisory Committee on Nutrition and Public Health England [57]. Mandatory folic acid fortification of non-wholemeal flour is scheduled for implementation in the UK by 2027, alongside recommendations for folic acid supplementation among women of reproductive-age [58]. In practice in the UK, however, both awareness and uptake of folic acid supplementation among women of reproductive-age remains suboptimal, reflecting longstanding limitations in implementation and prioritisation [59].

### 4.4. Potential Drivers of Observed Changes

Food consumption patterns have changed over the last two decades across Europe. In particular, mean intakes of meat and meat products, as well as fish and seafood, have declined among UK adults by approximately 39% and 29%, respectively (Table 4) [48,50]. Comparable reductions were smaller in France and the Netherlands. For example, in France, intakes decreased by 13% for meat (red meat, poultry and offal) and 13% for fish and seafood (fish, crustacea and molluscs) [51,52], whilst in the Netherlands, reductions were of the same order: 12% for meat and 6% for fish and seafood [44,45].

In the UK, these dietary shifts are likely to have contributed to declines in intakes of vitamin D, vitamin A, riboflavin (vitamin B2) and trace minerals including iron, selenium and iodine over the last two decades [60]. Although meat and meat products contain relatively low concentrations of vitamin D compared with oil-rich fish and some fortified foods, they remain an important contributor to vitamin D intake in the UK and Irish adults (Table 4). Importantly, meat provides both 25(OH)D_3_ and 1,25(OH)D_3_, downstream metabolites of vitamin D. 25(OH)D is known to have higher bioequivalence than cholecalciferol, which is used in food supplements and fortified food [61,62,63]. In contrast to trends observed in the UK and the Netherlands, meat and meat product consumption in Ireland remained relatively stable over the survey periods (Table 4). This stability may partly explain the comparatively improved intakes of vitamin D, vitamin A, riboflavin and iron among Irish adults relative to Dutch and British populations (Figure 2).

**Table 4:** Relative vitamin D contribution of food sources: intake and changes across the four European countries.

Country (National Food Survey Dates)	Food Source with Highest Contribution to Dietary Vitamin D [Latest Survey]	% Contribution of Total Dietary Vitamin D	Change in Mean Intake of Food Source; All Adults % [Intake g/Day BOLD Latest Survey]
NL (2008/10–2012/16)	Fats and oils	36	−9% [24 g; **22 g**]
IR (1997/9–2008/10)	Meat and meat products	30	2% [179 g; **183 g**]
FR (2006/7–2014)	Fish and seafood	31	−13% [31 g; **27 g**]
UK (2003–2020)	Meat and meat products	34	−39% [161 g; **99 g**]

Key: FR: France; IR: Ireland; NL: the Netherlands; UK: United Kingdom [44,45,46,47,48,50,51,52]. Reproduced from [64] licensed under CC BY 4.0.

### 4.5. Food Fortification

Interpretation of these country-level patterns should consider differences in fortification context, although these were not formally analysed in the present study. In Ireland, voluntary fortification expanded between 1997–1999 and 2008–2010, with the proportion of adults consuming fortified foods increasing from 67% to 82% and the contribution of fortified foods to daily energy intake rising from 4.6% to 8.4% [65]. Fortified staple categories such as breads, milk products and fat spreads contributed importantly to folate and other B-vitamin intakes, and previous FSAI reports have linked improved folate status in Ireland to voluntary folic acid fortification [65,66]. In the Netherlands, fortification is generally voluntary, but iodine intake has long been supported by iodised salt in bread and bakery products, while vitamins A and D are encouraged in plant-based fats through covenant-based arrangements [67,68]. In France, the picture is more limited and mixed: iodine intake reflects iodised salt use alongside dairy products, eggs and seafood [69], while vitamin D intake comes mainly from fish and dairy products, with some voluntary fortification of selected dairy products, cereals and oils rather than a broad mandatory programme [70]. By contrast, the UK relied largely on voluntary fortification during the study period, including vitamin D fortification of vegetable seed-oil spreads and cereals, while as stated above, mandatory folic acid fortification of non-wholemeal flour will only begin from the end of 2026 [58]. Taken together, these policy differences may have influenced background micronutrient exposure over time, but they should be interpreted as contextual factors rather than direct explanations of the intake changes observed here.

### 4.6. Role of Diet Quality and Food Supplementation

Although, theoretically, a healthy diet can provide sufficient quantities of most micronutrients, vitamin D is a notable exception, with requirements difficult to achieve from food sources alone. This is reflected in national supplementation recommendations across countries examined. In the UK, adults are advised to consider a daily supplement providing 10 µg vitamin D during the autumn and winter, with year-round supplementation recommended for groups at increased risk of deficiency [57]. In Ireland, adults aged 12–65 years are advised to take 15 µg daily during the extended winter, with year-round supplementation recommended for people with darker skin and during pregnancy [71]. The Netherlands adopts a more targeted approach, recommending 10 µg daily for groups including women aged 50–69 years, adults with darker skin or limited sunlight exposure and pregnant women, and 20 µg daily for adults aged ≥70 years [72]. In France, routine food supplementation is not recommended for all healthy adults; greater emphasis is placed on sunlight exposure and dietary sources, with supplementation considered on an individual basis where required [73]. These national recommendations provide important context for the consistently low dietary vitamin D adequacy observed across countries in the present analysis.

More broadly, European national dietary surveys demonstrate poor compliance with healthy eating guidelines, even in high-income countries, and suboptimal micronutrient intakes remain common throughout the life-course. For some population groups, achieving adequate micronutrient intake from diet alone may not be feasible due to socioeconomic, geographical and lifestyle factors. In this context, the appropriate use of micronutrient-containing food supplements, as defined in the Food Supplements Directive [38], may help bridge the gap between intake and requirements, thereby supporting nutritional safety, adequacy and health resilience. Despite this, the potential contribution of food supplements to maintaining adequate micronutrient status is often under-recognised within public health nutrition discourse, particularly where dietary intake distributions are shifting downward across multiple micronutrients simultaneously [74].

### 4.7. European Commission Regulatory Considerations and Policy Tensions

When considered alongside current proposals to change maximum permitted micronutrient levels in the EU, and subsequently food supplements and fortified foods as a consequence of the SPS EU-UK agreement [75], these findings highlight a potential emerging policy tension. Proposed European Commission MPLs for several micronutrients may, in some cases, be at odds with current practice. Whilst pre-existing MPLs protect individuals against excessive intake, proposed EC limits may simultaneously constrain the ability of food supplements to compensate for inadequate dietary intakes among vulnerable groups. This tension is particularly relevant in populations where dietary intakes of nutrients such as vitamin A, vitamin D, folate, calcium, magnesium, potassium, zinc, iron and iodine are already suboptimal. In such contexts, overly restrictive or misaligned MPLs may narrow the practical margin available for restoring nutritional adequacy and safety and supporting health resilience (Table 1).

### 4.8. Limitations of Proposed EC Maximum Permitted Micronutrient Levels (MPL) Model

Modelling approaches that aim to harmonise MPLs for food supplements and fortified foods across European Member States without accounting for substantial cross-country variation in baseline micronutrient intakes may therefore have limited public health relevance. This limitation may be further compounded by the adoption of a hazard-based risk assessment framework that prioritises high-end intake outliers over population intake distributions. Within the proposed EC model, additional conservative assumptions are applied, including the allocation of the residual intake (UL minus P95) on a 50:50 basis between food supplements and fortified foods, followed by extrapolation to children using high-end energy intake data (p95) and an estimation of fortified food contributions in younger age groups.

The present findings underscore the limitations of such an approach by demonstrating substantial heterogeneity between countries in the direction and magnitude of temporal change. Together, these results suggest that theoretical, highly standardised regulatory models may be insufficiently responsive to population-specific nutritional needs and could potentially exacerbate existing micronutrient insufficiencies in some populations.

### 4.9. Implications for Nutritional Risk Assessment

From a regulatory perspective, this study highlights the importance of balancing two complementary dimensions of nutritional safety: protecting against excessive intake at the upper end of the distribution and defending against inadequacy at the lower end. Policies designed primarily to control potential overexposure using hazard-assessment risk models may inadvertently narrow the practical capacity to address emerging inadequacies if dietary intakes are not simultaneously considered. The present analysis therefore highlights the importance of incorporating full distributional intake data into nutritional surveillance and regulatory risk assessment. Reliance on mean intakes or highest percentile values alone may obscure emerging vulnerabilities among lower intake groups, particularly where declines occur unevenly across the population. Monitoring trends across the full intake distribution, including lower percentiles, may provide a more comprehensive and sensitive indicator of changes in nutritional risk.

### 4.10. Study Limitations

Despite the strengths of this cross-percentile examination using EFSA methodologically aligned national dietary surveys, some inherent limitations should be acknowledged and discussed. First, although the study design enabled assessment of temporal change, the surveys were not participant-matched and therefore do not represent prospective follow-up of the same individuals. The use of observed, unadjusted micronutrient intakes across percentiles may have resulted in day-to-day variation and could widen the intake distribution, especially at the lower and upper percentile tail ends. Because our secondary analyses were restricted to publicly available summary statistics across percentiles, individual level data were not available. This limited the ability to undertake additional statistical modelling or adjustment and should be considered when interpreting the findings. Additionally, within-country comparisons across survey years should be interpreted as repeated cross-sectional population estimates, rather than individually informed longitudinal trajectories.

Although the analysis was constrained by the timing of the available national dietary surveys, with countries not collecting data at identical time points within the 1999–2020 study period, the relatively long intervals between survey periods may have facilitated the detection of changes in micronutrient intakes within individual countries. Marked changes in dietary patterns and eating habits may have occurred over these extended intervals and could have contributed to the observed changes in micronutrient intake (Table 4). As the study was not designed to make direct inter-country comparisons, differences in survey timings do not affect the within-country temporal analyses, but should be considered when interpreting the findings collectively.

Similarly, there was a lack of national harmonisation in defining distribution tails, for example, the use of the p2.5 and 97.5 for the UK, versus p5 and p95 for the other countries. However, since identical published metrics were compared between repeated survey waves within each country, the observed temporal trends remain informative regarding changes within population intake distributions.

Finally, interpretation of intake adequacy relative to EARs or LRNIs should be considered cautiously. Individuals with intakes at the lower end of the population distribution do not necessarily have lower physiological requirements, and therefore low intake alone cannot be assumed to equate directly to inadequacy at the individual level. While dietary reference values are valuable for population assessment, they may provide an incomplete framework for understanding nutritional vulnerability, particularly where broader downward shifts in intake distributions occur across multiple micronutrients simultaneously. The intake adequacy measures were assessed by comparing intakes to EARs, aMIVs or RNIs, since the publicly available national surveys did not provide individual-level datasets.

### 4.11. Evidence Gaps and Future Research Recommendations

Aside from the UK and Ireland, the distinct lack of cross-country micronutrient comparisons in Europe could be transformed by use of harmonised dietary assessment methods, including harmonised food consumption data. Future research incorporating biomarker data and functional health outcomes would also allow correlations to be made between micronutrient intake and health resilience. Further research should identify population groups most vulnerable to micronutrient inadequacy and examine the socioeconomic factors contributing to inequalities in micronutrient intake. This would strengthen the assessment of nutritional risk at both individual and population levels.

## 5. Conclusions and Public Health Implications

We explored the hypothesis that micronutrient intakes in European adults have not improved over time and remain inadequate relative to established dietary reference values. Across four European countries, mean and lower-tail intakes of several key micronutrients, including vitamins D, A, riboflavin and folate, together with calcium, iron and iodine, declined substantially, by up to 50% in some population groups. These declines were frequently most pronounced among those with the lowest intakes, suggesting widening distributional disparities in micronutrient exposure. Cross-percentile reduction patterns in the UK were observed for intakes of folate and iodine. Although some improvements were observed and were broadly consistent with national fortification practices and changes in food consumption patterns, inadequacies remained widespread across population groups.

Despite country-specific heterogeneity, inadequacies remained widespread, including vitamin D in both sexes across all countries, iron in women across all countries, potassium in UK women, selenium in both sexes across all countries, aside from UK men, and copper in Irish women. Folate intake among UK women and vitamin A consumption in France remained marginal.

Overall, our findings indicate that contemporary European micronutrient nutrition is characterised not only by persistent inadequacies, but also by declining intakes and widening disparities in intake distributions. While micronutrient inadequacy in Europe is well recognised, our study extends existing evidence by examining temporal changes across intake percentiles, thereby providing insight into how population distributions change over time. To our knowledge, comparable intra-national analyses of long-term micronutrient intake trends across percentiles have not previously been reported in European adult populations.

The observed patterns suggest a progressive erosion of nutritional safety margins among population groups already at greatest risk of inadequacy, with potential implications for safety risks and population health resilience (Table 1). These trends may have implications for public health resilience, particularly where habitual intakes are close to, or below reference levels. Our findings support continued investment in harmonised, prospective national dietary survey surveillance across Europe, including reporting across intake distributions rather than reliance solely on population means.

From a nutrient policy perspective, persistent inadequacies and declining intakes may warrant ongoing discussions concerning the regulation of food supplements and fortified foods, including harmonised Maximum Permitted Levels (MPLs). While protecting consumers from excessive micronutrient intakes has been and remains a vital regulatory objective, consideration should also be given to the prevalence of inadequacy, existing intake distributions and the nutritional requirements of vulnerable populations groups. Regulatory frameworks that balance safety with nutritional adequacy may be better positioned to support population nutritional safety and resilience in the context of evolving European dietary behaviours and widening inequalities in micronutrient intakes.

## Figures and Tables

**Table 1:** Acute and chronic consequences of systemic homeostatic impairment due to micronutrient insufficiency, organised by body system. Cross-references indicate shared inflammatory pathways between systems ^†^.

System	Acute Homeostatic Impairment and Selected Pathophysiological Effects	Chronic Homeostatic Impairment and Selected Clinical Effects
**Cardiometabolic**	Loss of nutrient balance sensing in enteroendocrine, liver, and adipose cells, impairing production of homeostatic and metabolic signalling molecules. **Physiological consequence:** Dysregulated insulin activity Impaired triglyceride breakdown and low-density lipoprotein clearance Disrupted satiation/hunger signalling via gut-brain crosstalk Altered thermogenesis Impaired microvascular endothelial integrity **Vitamins**: B vitamins (B2, B9 (folate)) and vitamin D **Minerals:** calcium, magnesium, potassium, phosphorus, iron, zinc, selenium and chromium	**Chronic immune activation and persistent low-grade inflammation:** Obesity; pre-diabetes; type 2 diabetes mellitus; polyendocrine metabolic ovarian syndrome (PMOS) Atherosclerosis and cardiovascular disease Metabolic dysfunction–associated steatotic liver disease (MASLD) Cachexia
**Brain and Nervous System**	Neurological impairment of defence, regulation, and maintenance in glial, macrophage, Schwann cells, and the vasa nervorum. **Physiological consequence:** Dysregulated response to stress, infection, and inflammatory challenge Impaired neural remodelling, plasticity, and adaptive healing Reduced vagal tone and impaired autonomic regulation **Vitamins:** B vitamins (B1, B6, folate (B9), B12), β-carotene, and vitamins D, E and K **Minerals:** Calcium, potassium, magnesium, phosphorus, zinc and copper	**Chronic immune activation and persistent neuroinflammation:** Impaired learning, memory, cognition, and motor function Disrupted emotional and behavioural processes Cognitive decline and dementias including Alzheimer’s disease *(See also: Cardiometabolic—shared inflammatory pathways)*
**Reproductive**	Disruption of key physiological processes involved in reproduction. **Physiological consequence:** Dysregulated ovulation and spermatogenesis Impaired maternal-foetal interface in pregnancy Disrupted mammary gland development **Vitamins:** B vitamins (B2, B9 (folate), B12), and vitamins C, D, and E and β-carotene **Minerals:** Calcium, magnesium, iron, iodine, selenium, zinc and copper	**Chronic immune activation and persistent inflammation:** Male and female infertility PMOS; dysmenorrhea Poor pregnancy outcomes (pre-eclampsia; preterm birth; postnatal complications; maternal postnatal depression)
**Immune**	Breakdown of epithelial barrier function; impairment of innate and adaptive immune responses; loss of inflammatory homeostasis. **Physiological consequence:** Increased infection susceptibility (bacterial, viral, fungal) Increased translocation of microbial products across organs including the gastrointestinal tract, amplifying systemic inflammation Poor vaccination responses **Vitamins:** B vitamins (including B2, B6 and B9 (folate)), and vitamins A, C, D and E **Minerals:** Magnesium, iron, selenium, zinc and copper	**Weakened immunity:** Increased susceptibility to and severity of infections Impaired vaccination response; increased hospitalisations Increased antibiotic use contributing to antimicrobial resistance **Chronic low-grade inflammation:** Atherosclerosis, cardiovascular disease, metabolic disease (type 2 diabetes, MASLD), cognitive decline and Alzheimer’s disease, sarcopenia, some cancers *(See also: Cardiometabolic; Brain and Nervous System; Musculoskeletal; Gastrointestinal and Reproductive)* **Chronic high-grade inflammation:** Autoimmune conditions (e.g., rheumatoid arthritis, multiple sclerosis, inflammatory bowel disease, endometriosis)
**Musculoskeletal**	Dysregulation of bone and muscle maintenance and repair. **Physiological consequence:** Impaired bone cell density regulation; dysregulated osteoclast/osteoblast activity End-plate bone hypertrophy during growth Dysregulated repair of skeletal muscle microtears and post-trauma regeneration **Vitamins:** B vitamins (B12), and vitamins C, D, E and K **Minerals:** Calcium, phosphorus, magnesium, selenium, zinc and copper	**Chronic innate immune activation and persistent inflammation:** Osteomalacia; nutritional rickets (children); osteoporosis Muscle weakness; sarcopenia; cachexia; fibromyalgia *(See also: Immune—shared inflammatory pathways)*
**Gastro-intestinal**	Dysregulation of the epithelial barrier and damage to the mucosal lining. **Physiological consequence:** Weakened barrier function; increased susceptibility to intestinal infections Increased gut permeability; diarrhea, vomiting; fluid loss Impaired mucosal immunity Greater susceptibility to inflammatory damage Dysbiosis **Vitamins:** B vitamins, and vitamins C, D and E **Minerals:** Magnesium, iron, selenium, zinc and copper	Dysregulation of epithelial integrity increasing permeability, decreasing efficiency, motility and protective properties **Physiological consequence:** Loss of integrity with villous atrophy, mucin impairment, tight junction breakdown and increased permeability Chronic cytokine-driven inflammation altering tissue architecture and abnormal elasticity, mucosal destruction Dysbiosis leading to destabilised biofilm-mucus-epithelium interface Greater risk of infections and autoimmune dysfunction **Chronic immune activation, persistent inflammation and loss of tissue integrity:** Leaky gut; Irritable Bowel Syndrome (IBS) Inflammatory Bowel Disease; auto-immune enteropathy *Bidirectional cross-talk from the gastrointestinal- tract (enteroendocrine, lymphoid and microflora) to all other body systems*

^†^ Examples are selective and non-exhaustive. Abbreviations: DM, diabetes mellitus; MASLD, metabolic dysfunction-associated steatotic liver disease; PMOS, Polyendocrine Metabolic Ovarian Syndrome.

**Table 2:** National European Food Surveys: summary of characteristics.

Country	Survey; Publication Date *	Survey Methodology; Latest (P): Prospective	Participants in Each Survey (*n*)	Number of Years Between Surveys [Latest Survey: Total Number of Nutrients Assessed (V/M)]
NL	DNFCS 2012; 2021	(P) 2-d non-consecutive diary	1540; 1747	9 yrs; [18 (9/9)]
IR	NSIFSC 1997; NANS 2010	(P) 4-d consecutive semi-weighed diary	1379; 1274	13 yrs; [16 (10/6)]
UK	NDNS: 2003; 2014; 2020	(P) 4-d non-consecutive diary	1724; 2000; 3558	16 yrs; [12 (5/7)]
FR	INCA2 2006; INCA3 2014	(P) 3-d non-consecutive recall	1918; 2121	8 yrs; [15 [9/6)]

* Survey Field Work Years (Start-Finish): NL DNFCS 2012 (2012–2016) [44]; NL DNFCS 2021 (2019–2021) [45]; IR NSIFSC 1997 (1997–1999) [46]; IR NANS 2010 (2008–2010) [47]; UK NDNS 2003 (2000–2001) [48]; UK NDNS 2014 (2008–2012) [49]; (UK NDNS 2020 (2016–2019) [50]; FR 2006 INCA2 (2006–2007) [51]; FR INCA3 2014 (2013–2014) [52] Key: d: day; FR: France; IR: Ireland (North and South); n: number; NL: the Netherlands; M: minerals; UK: United Kingdom; V: vitamins. DNFCS: Dutch National Food Consumption Survey. NSIFSC: North/South Ireland Food Consumption Survey. NANS: National Adults Nutrition Survey. NDNS: National Diet and Nutrition Survey.

**Table 3:** Integrated summary of notable European micronutrient intake shifts and adequacy across distributions with public health interpretation ° ^†^.

Nutrient	Mean Intake Change Notable (≥/10%)	Mean Adequacy (EAR/aMIV)	Lowest Percentile Change Notable (>/10%)	Lowest Percentile Adequacy (LRNI)	Highest Percentile Change Notable (>/10%)	Highest Percentile Adequacy (RNI)	Public Health Interpretations
Vitamin D	Mixed: UK decline (−14% men); FR increases (21–26%)	Low across all countries (25–34% RNI)	Largest declines (UL: −40 to −50%; IR/NL: −14 to −22%)	Very low (3–12% LRNI)	Small mixed changes; some improvement (e.g., UK men +10%)	Still below RNI in most groups (NL: 49–60%; IR: 71–85%; UK men 102%)	Persistent inadequacy across the distribution; most severe at lower percentile
Vitamin A	Declines in FR/UK (−10 to −30%)	Mean intakes met EAR	Consistent declines (~−10 to −20%)	Below LRNI in UK/IR; NL above	Declines in some groups (FR−28%; UK~ −13%)	Well above RNI	Lower-percentile inadequacy despite adequate mean and upper tail intakes
Folate	UK declines (−16 to 23%); IR increases (+17–18%)	Mean intakes met EAR	UK declines greatest (−25 to −28%)	UK women below LRNI (76%)	Increases in IR (+29 to 35%); declines in UK (−13 to −16%)	Above RNI	Divergence across whole intake distribution; vulnerability concentrated in low-intake groups
Riboflavin (B2)	UK declines (−13 to −19%)	Mean intakes met EAR	Moderate declines in some groups	NL, IR and UK women below LRNI (63–88%)	Mixed: increases in IR (+12–25%); declines in UK (−15 to −16%)	Above RNI (up to 181%)	Mean adequacy masks lower percentile shortfalls
Vitamin B12	Mean increase in IR (+23%)	Mean intakes met EAR	Small declines in some groups	Above LRNI (160–180%)	Stable/no major changes	Very high (>700% RNI in some groups)	Adequate across all percentiles
Other B vitamins/Vitamin C	Moderate declines in some groups (−10 to −17%)	Generally met EAR	Some moderate declines (>10%)	Generally adequate	Some increases in IR (e.g., vitamin C +16%)	Above RNI	Broad shifts but limited adequacy concerns
Iron	Declines in FR/UK (−12 to −24%)	Women cross countries below EAR (77–92%); men above	Moderate declines (~−12%)	Women below LRNI (45–75%); men above	Increases in IR	Most groups above RNI; Dutch women below (89%)	Persistent sex-specific vulnerability, particularly in women
Calcium	Some mean declines (−12%)	Mean intakes broadly adequate	Declines in UK (−10 to −21%)	Below LRNI in UK/IR groups	Increased in IR (+10 to 11%)	Above RNI	Lower-intake percentile inadequacy despite adequate upper-tail intakes
Magnesium	Some increases (FR +16%)	Mean intakes met EAR	Moderate declines (~ −13%)	Below LRNI in some UK/IR groups	Increase in UK women (+11%)	Above RNI	Lower-tail vulnerability masked by adequate means
Potassium	Mostly stable	UK women below aMIV (93%)	Lower-tail shortfalls	UK women 54% LRNI	No major changes	Above RNI in upper intake percentile groups	Vulnerability concentrated at lower percentile intakes
Zinc	Small declines	Mean intakes broadly adequate	Moderate declines (UK men −13%)	UK below LRNI (76–78%)	Increase in UK women (+12%)	Above RNI	Lower-tail deterioration despite adequate upper-tail intakes
Selenium	Below aEAR in UK/NL (85–97%)	Borderline inadequate	Further lower-tail decline (−15%)	Below LRNI (43–78%)	Increase in UK women (+13%)	Marginally above RNI (~114–115%)	Chronic inadequacy in lower and mean intakes
Iodine	Mean decline in UK (−22% men; −11% women)	Borderline adequacy	Large declines (−28 to −41%; UK)	UK below LRNI (60–67%)	No major decline; some increases in France (+18 to +19%)	Around RNI	One of the clearest examples of lower-tail deterioration
Copper	Mostly stable	Near adequacy	Ireland decline (−23%)	Dutch below LRNI (42–75%)	No major changes	Above RNI	Country-specific vulnerability confined to lower tail

Key: ° Notable intake changes defined as ≥10%. ^†^ Countries: the Netherlands (NL), Ireland (IR), France (FR) and the United Kingdom (UK) Mean adequacy was assessed against Estimated Average requirements (EARs), or approximated Median Intake Values MIVs (aMIVs) where EARs were not available (see Materials and Methods for Calculation Method); lowest intake percentile assessed against Lower Reference Nutrient Intakes (LRNIs or RNI for vitamin D), and highest dietary intakes assessed against Reference Nutrient Intakes (RNIs).

## Data Availability

The original contributions presented in this study are included in the article/Supplementary Material. Further inquiries can be directed to the corresponding author.

## Supplementary Materials

The following supporting information can be downloaded at: https://www.mdpi.com/article/10.3390/nu18172860/s1, Figure S1: Percentage change in lowest-tail percentile vitamin intakes between previous and latest national food surveys across selected European countries (combined dataset: NL, IR and UK); Figure S2: Percentage change in mean vitamin intakes between previous and latest national food surveys across selected European countries (combined dataset: NL, IR, FR and UK); Figure S3: Percentage change in highest-tail percentile vitamin intakes between previous and latest national food surveys across selected European countries (combined dataset: NL, IR and UK); Figure S4: Percentage change in lowest-tail percentile mineral intakes between previous and latest national food surveys across selected European countries (combined dataset: NL, IR and UK); Figure S5: Percentage change in mean mineral intakes between previous and latest national food surveys across selected European countries (combined dataset: NL, IR, FR and UK); Figure S6: Percentage change in highest-tail mineral intakes between previous and latest national food surveys across selected European countries (combined dataset: NL, IR and UK).

## Abbreviations

The following abbreviations are used in this manuscript:

EAR	Estimated Average Requirement
aMIV	approximated Median Intake Value
LRNI	Lower Reference Nutrient Intake
RNI	Reference Nutrient Intake
MPL	Maximum permitted level
UL	Safe tolerable upper level
EFSA	European Food Safety Authority
