# Supplementary material for: Nutritional Safety and Health Resilience in Europe: Declining Micronutrient Intakes, Widening Distributional Disparities and Persistent Inadequacies Revealed from National Dietary Surveys
Source: Nutrients. 2026 Sep 2;18(17):2860. doi: 10.3390/nu18172860 (PMC13567139; doi:10.3390/nu18172860)
Supplement: Supplementary file 1 [file nutrients-18-02860-s001.zip › nutrients-4468568-supplementary.pdf]

Supplementary Materials

Nutritional Safety and Health Resilience in Europe: Declining Micronutrient Intakes, Widening Distributional Disparities and Persistent Inadequacies Revealed from National Dietary Surveys

Samantha Christie [1,*], Adrian F. Gombart [2], Thomas R. Hill [3,4], Tao You [5,6,7] and Philip C. Calder [8,9]

Figure S1: Percentage change in lowest-tail percentile vitamin intakes between previous and latest national food surveys across selected European countries (combined dataset: NL, IR and UK) [*†θ]

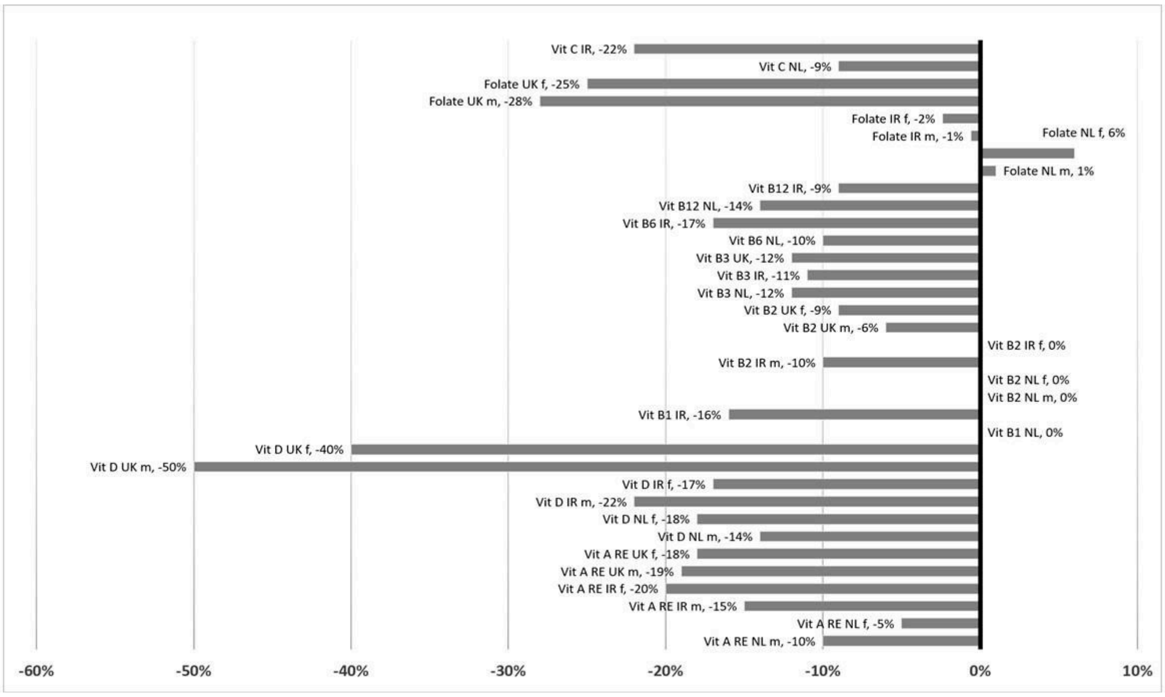

Key: *Food-only datasets; †: vitamin A as Retinol Equivalents (RE); θ: lowest-tail: p2.5 (UK) or p5; IR: Ireland; NL: the Netherlands; UK: United Kingdom; Vitamin B2: riboflavin; folate (Vitamin B9); Vitamin C not assessed separately for men and women

Figure S2: Percentage change in mean vitamin intakes between previous and latest national food surveys across selected European countries (combined dataset: NL, IR, FR and UK) *†θ

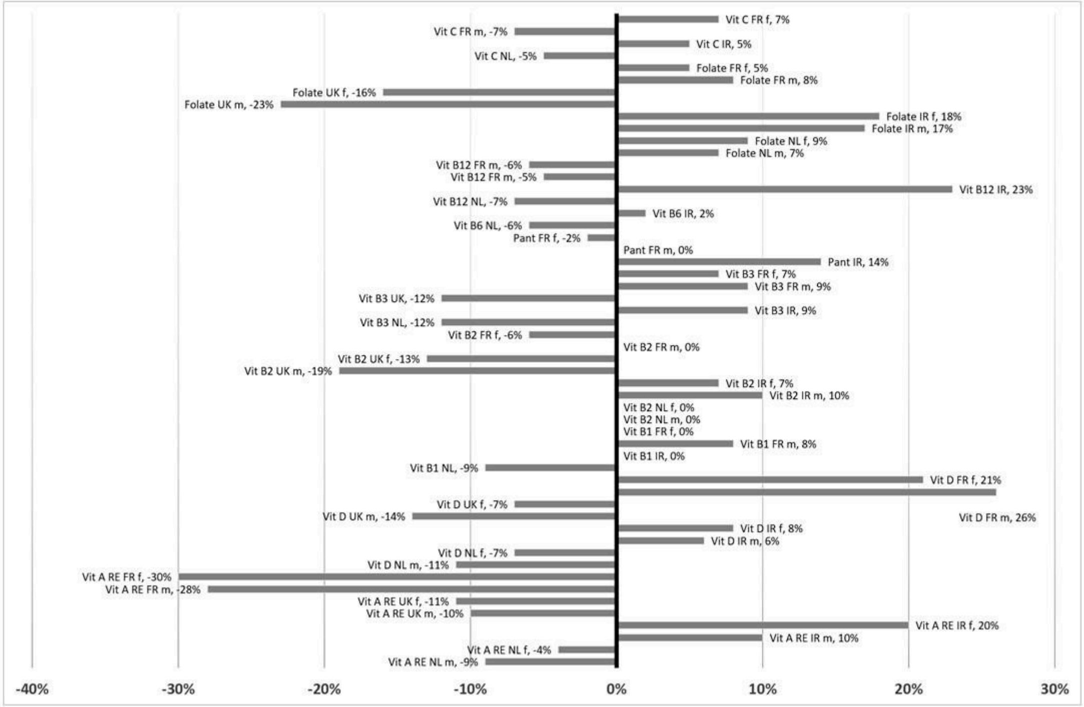

Key: *Food-only datasets; †: vitamin A as Retinol Equivalents (RE); θ: FR mean datasets only; FR: France; IR: Ireland; NL: the Netherlands; UK: United Kingdom; Vitamin B2: riboflavin; folate (Vitamin B9); Vitamin C not assessed separately for men and women

Figure S3. Percentage change in highest-tail percentile vitamin intakes between previous and latest national food surveys across selected European countries (combined dataset: NL, IR and UK) *†θ

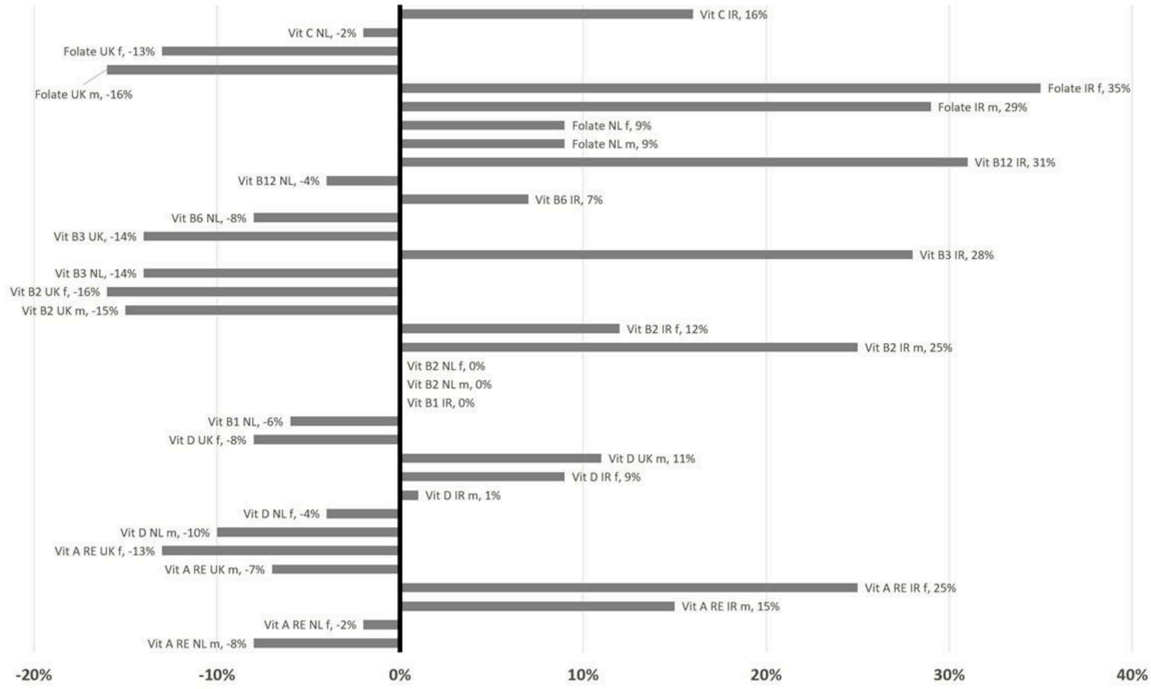

Key: *Food-only datasets; †: vitamin A as Retinol Equivalents (RE); θ: highest-tail: p97.5 (UK) or p95; IR: Ireland; NL: the Netherlands; UK: United Kingdom; Vitamin B2: riboflavin; folate (Vitamin B9); Vitamin C not assessed separately for men and women

Figure S4. Percentage change in lowest-tail percentile mineral intakes between previous and latest national food surveys across selected European countries (combined dataset: NL, IR and UK) *†θ

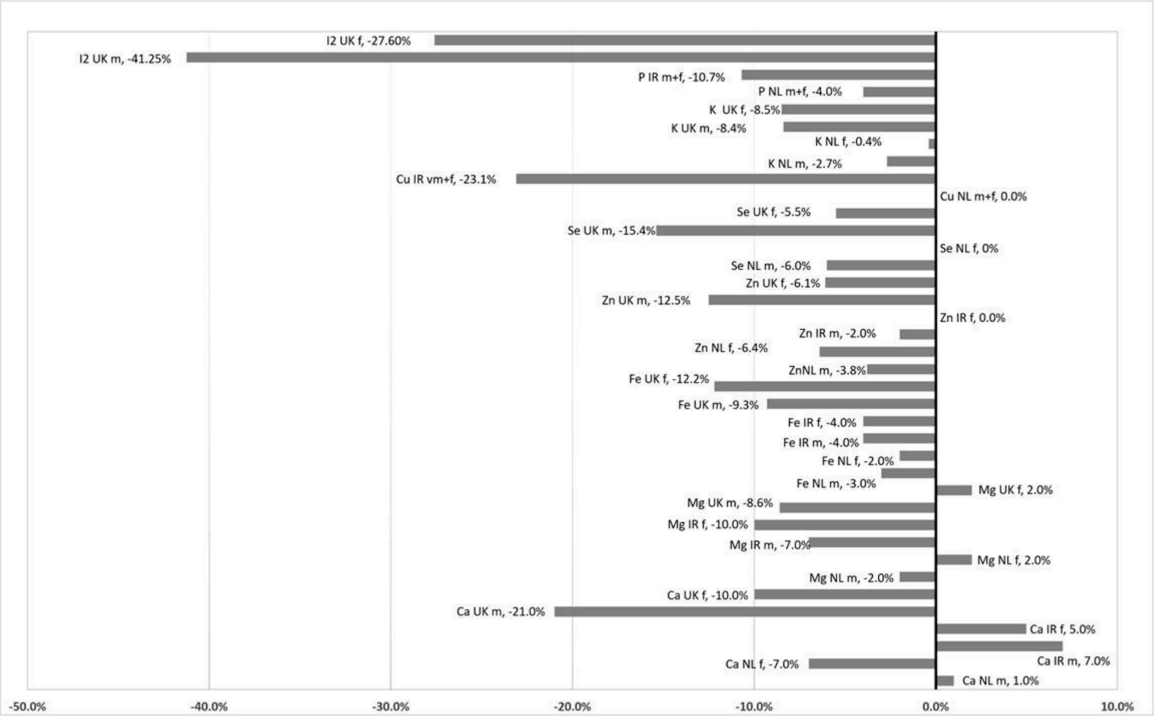

Key: *Food-only datasets; θ: lowest-tail intake p2.5 (UK) or p5; Ca: calcium; Fe: iron; IR: Ireland; M: males; f: females; I2: iodine; K: potassium; Mg: magnesium; NL: the Netherlands; P: phosphorus (m and f combined); Se: selenium; UK: United Kingdom; Zn: zinc

Figure S5. Percentage change in mean mineral intakes between previous and latest national food surveys across selected European countries (combined dataset: NL, IR, FR and UK) *†

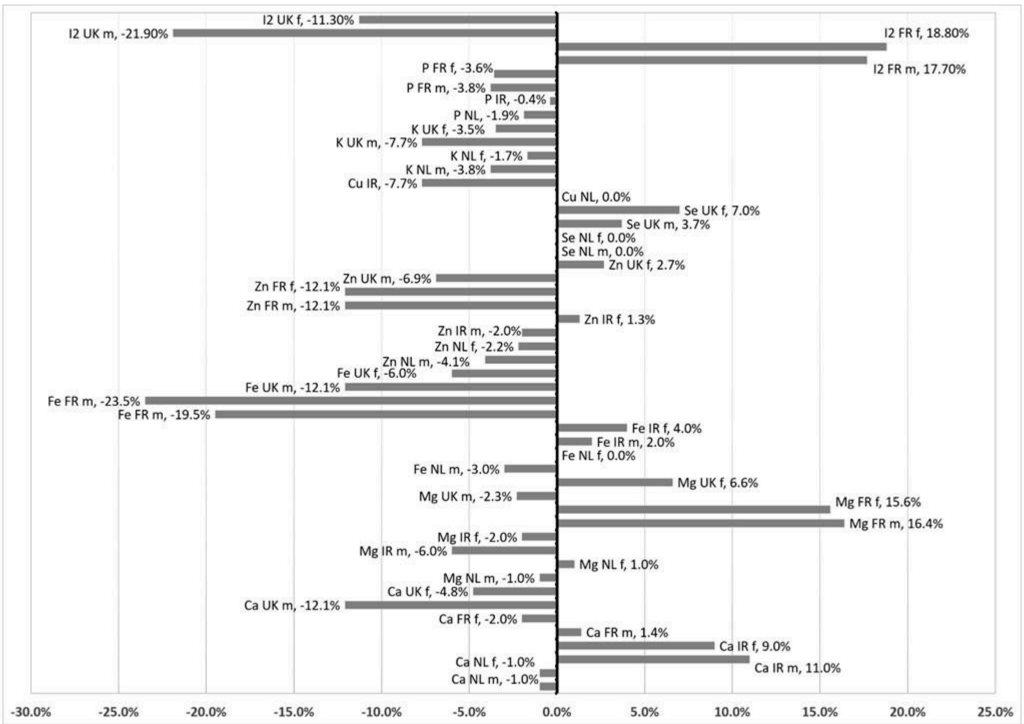

Key: *Food-only datasets; Ca: calcium; Fe: iron; IR: Ireland; M: males; f: females; I2: iodine; K: potassium; Mg: magnesium; NL: the Netherlands; P: phosphorus (m and f combined); Se: selenium; UK: United Kingdom; Zn: zinc

Figure S6. Percentage change in highest-tail mineral intakes between previous and latest national food surveys across selected European countries (combined dataset: NL, IR and UK) *†

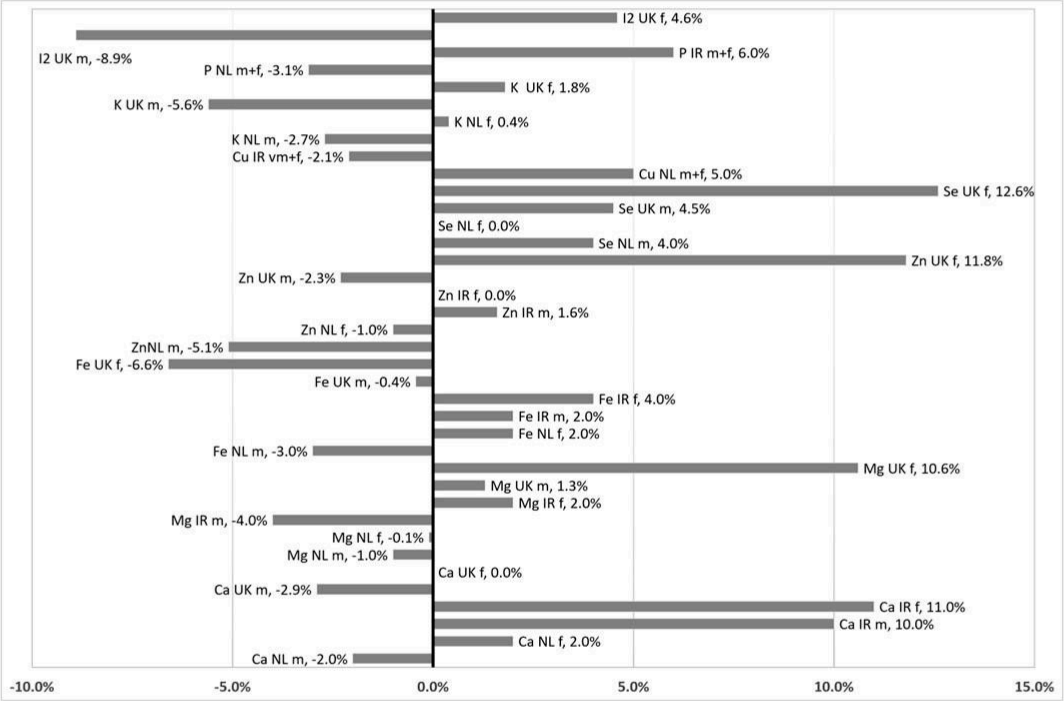

Key: *Food-only datasets; θ: highest-tail intake p97.5 (UK) or p95; Ca: calcium; Fe: iron; IR: Ireland; M: males; f: females; I2: iodine; K: potassium; Mg: magnesium; NL: the Netherlands; P: phosphorus (m and f combined); Se: selenium; UK: United Kingdom; Zn: zinc
